# Symbiotic soil fungi mitigate nitrogen-driven methane emissions in an experimental grassland

**DOI:** 10.3389/fmicb.2026.1815239

**Published:** 2026-06-10

**Authors:** Yangyang Jia, Wan Duan, Hui Wang, Wenshuo Li, Wenli Cao, Fangwei Zhang, Weiguo Liu

**Affiliations:** 1 College of Ecology and Environment, Xinjiang University, Urumqi, China; 2 Key Laboratory of Oasis Ecology, Xinjiang University, Urumqi, China

**Keywords:** arbuscular mycorrhizal fungi, carbon cycle, global climate change, methane emission, nitrogen deposition, non-waterlogged condition

## Abstract

Methane (CH_4_) emission is a critical natural process contributing to atmospheric greenhouse gas accumulation and constitutes an important component of global carbon cycling. Arbuscular mycorrhizal (AM) fungi play vital roles in regulating greenhouse gas emissions and participate in soil carbon cycling. However, the understanding of the functions and regulatory patterns of AM fungi in regulating soil CH_4_ emissions remains equivocal, particularly under non-waterlogged conditions accompanied by increased nitrogen (N) deposition. To fill this critical knowledge gap, this study dynamically monitored soil CH_4_ fluxes spanning three plant growth seasons of the presence/absence of AM fungi under increased N deposition in an experimental grassland. The study found that increased N deposition stimulated soil CH_4_ emissions, but this response was only detected in the absence of AM fungi. AM fungi exhibited a significant association with soil CH_4_ emissions, and these correlative patterns were dependent on N deposition levels. Specifically, AM fungi significantly mitigated the stimulatory effects of high N deposition on CH_4_ emissions, but AM fungi were correlated with elevated soil CH_4_ emissions under low N deposition, likely in association with higher plant community diversity. Furthermore, plant community Shannon–Wiener diversity acted as a key factor that was interactively modulated by increased N deposition and AM fungi, showing a statistical association with soil CH_4_ emissions. These findings provide experimental evidence that AM fungi are involved in regulating soil CH_4_ emissions in an N deposition-dependent manner, and further highlight the close statistical linkage between plant community diversity and soil CH_4_ emissions. Meanwhile, these results underscore the necessity of future study to quantify changes in soil microbial communities associated with CH_4_ production and oxidation processes induced by AM fungi, contributing to informing evidence-based policies for mitigating global warming and ensuring sustainable ecosystem management under ongoing climate change.

## Introduction

Methane (CH_4_) is the second most powerful greenhouse gas after carbon dioxide (CO_2_), contributing approximately 20% of global radiative forcing associated with climate warming (IPCC, 2007). Traditionally, CH_4_ emissions have been viewed as predominantly occurring under strictly anaerobic conditions, regulated primarily by soil moisture and temperature. Accordingly, most studies have mainly focused on rice paddies and wetland ecosystems (Jang et al., 2011; Tong et al., 2025; Wang P. et al., 2025; White et al., 2023). Grassland soils are widely recognized as major sinks of CH_4_, with an estimated annual uptake of 1.49 kg CH_4_ ha^−1^, playing a critical role in mitigating global warming potential (Dutaur and Verchot, 2007; Li et al., 2025). Anthropogenically increased nitrogen (N) deposition has been widely documented to substantially alter soil CH_4_ emissions in terrestrial ecosystems (Chen et al., 2022; Jang et al., 2011; Rafalska et al., 2023). Compared with the well-documented impacts of N deposition on biodiversity, it remains poorly understood how increased N deposition regulates soil CH_4_ emissions in grassland ecosystems (Zhang et al., 2024; Zuccarini et al., 2023). Filling this knowledge gap is essential for improving predictions of how global climate change affects C cycling linked to soil CH_4_ emissions.

Increased N deposition drives profound changes in soil physicochemical properties, plant community composition, and soil microbial communities, all of which jointly regulate soil CH_4_ emissions (Liu and Greaver, 2009; Petro et al., 2023; Piñeiro et al., 2023). Both nitrate (NO_3_^−^-N) and ammonium (NH_4_^+^-N) inputs directly affect CH_4_ production and oxidation processes (Bodelier and Laanbroek, 2004). Specifically, increased soil NO_3_^−^-N suppresses methanogenesis by increasing soil redox potential, while NH₄^+^-N inhibits CH₄ oxidation through competitive inhibition of methane monooxygenase (Kollah et al., 2023; Peng et al., 2019). Moreover, chronic N deposition induces soil acidification, which further modifies soil microbial functional traits beyond direct nutrient effects (Hu et al., 2024; Tang et al., 2024). Additionally, increased N deposition alters plant community biomass and diversity, reshaping root exudation and labile C supply for methanogens, thereby indirectly regulating soil CH_4_ production (Wilkins et al., 2022; Liu et al., 2021; Ma et al., 2022). Together, these direct and indirect pathways make the response of grassland CH₄ emissions to increased N deposition complex and context-dependent.

Arbuscular mycorrhizal (AM) fungi are key components of soil microbial communities, forming mutual symbiosis with over 80% of terrestrial plants (over 250,000 plant species; Smith and Read, 2008; Tedersoo et al., 2014; van der Heijden et al., 2015). AM fungi are of immense significance for ecologists due to their substantial contributions to soil organic C formation in the context of ongoing global change (Martin and van der Heijden, 2024; Tedersoo et al., 2020). Host plants allocate 5–30% of photosynthates to AM fungal symbionts, a flux equivalent to approximately 36% of global annual CO_2_ emissions from fossil fuels (Hawkins et al., 2023). Surprisingly, despite their ecological importance, the roles of AM fungi in mediating soil CH_4_ emissions remain rarely investigated, especially under increased N deposition (Martin and van der Heijden, 2024). Pioneering studies have indicated that AM fungi can suppress soil CH_4_ emissions in rice paddies, meadow grasslands, and desert ecosystems, with potential effects linked to altered soil NO_3_^−^-N contents and plant-microbe interactions (Zhang et al., 2017; Kang et al., 2020; Yue et al., 2020). These findings shed light on the roles of AM fungi in regulating soil CH_4_ emissions; however, existing evidence remains fragmented. Notably, few studies have explicitly addressed whether and how AM fungi modulate the magnitude and direction of increased N deposition effects on soil CH_4_ emissions in non-waterlogged conditions, leaving a critical knowledge gap in existing global change ecology.

Grasslands are historically regarded as net CH_4_ sinks (Chang et al., 2021; Ernst et al., 2022; Wang X. et al., 2025). Increased N deposition is thought to weaken CH_4_ sink strength or even shift grasslands toward net CH_4_ sources (Liu and Greaver, 2009). Most relevant evidence derives from short-term monitoring or single-point observations, with limited integration of plant community traits and mycorrhizal regulation (Jang et al., 2011; Trevathan-Tackett et al., 2025; White et al., 2023; Zhang et al., 2017). Importantly, how plant biomass and community diversity mediate CH_4_ flux under increased N deposition, and whether AM fungi act as a critical regulator in this framework, remain unclear. In particular, the magnitude, direction, and underlying mechanisms of AM fungal regulation of N deposition-induced changes in soil CH_4_ emissions remain unresolved. To address these limitations, this study simulated a typical European grassland system, continuously monitored soil CH_4_ fluxes over 7 days following an N pulse across three plant growth periods. This study specifically focused on the regulatory role of AM fungi in governing the responses of soil CH_4_ emissions to increased N deposition. The study hypothesized that (1) increased N deposition stimulates soil CH_4_ emissions by increasing mineral N availability and inducing soil acidification; (2) AM fungi mitigate the stimulatory effects of increased N deposition on soil CH_4_ emissions; and (3) AM fungi may enhance soil CH_4_ emissions by promoting plant community biomass and diversity under low N deposition.

## Materials and methods

### Experimental design

To test the interactive effects of increased N deposition and AM fungi on soil CH_4_ emissions, this study conducted a greenhouse experiment simulating one typical European grassland community from December 2018 to July 2019. This typical European grassland community consisted of 10 plant species, including four grass species, four forb species, and two legume species, with a total of 33 plant individuals, and detailed plant community information was shown in Jia et al. (2021). This study adopted a two-factor full design with N deposition (such as ambient N deposition, AN; and increased N deposition, IN) and AM fungi (such as without AM fungi, NM; and with AM fungi, M), yielding four treatments, each replicated eight times, resulting in a total of 32 experimental microcosms. For the growth substrate, this study collected soils from a calcareous grassland in Nenzlingen, Switzerland, which had an average soil pH of 8.1. The soil was sieved to <2 mm size, sterilized by autoclaving in two cycles at 121 °C for 90 min, and mixed with autoclaved quartz sand at a ratio of 1:1.25 to reduce organic matter concentration. The mixture was then allowed to stand for 1 month before use (Jia et al., 2021). The experimental system was established in pots pre-sterilized with ethyl alcohol. The bottom of each microcosm was lined with a white mesh and covered with 400 g of autoclaved sand (3 cm height). Each microcosm was then filled with 3.49 kg (dry weight) of the soil–sand mixture.

For the AM fungi treatment (M), each microcosm received 100 g of inoculum containing four AM fungal species–*Claroideoglomus claroideum*, *Funneliformis mosseae*, *Glomus diaphanum*, and *Rhizoglomus irregularis–*which are commonly found in European grasslands (Oehl et al., 2010). The inoculum was applied 10 cm below the soil surface to ensure early colonization of seedlings by AM fungi. For the without-AM fungi treatment (NM), 100 g of autoclaved AM fungal inoculum was added at the same depth. The entire 33-week experimental period was divided into three plant growth seasons (periods 1, 2, and 3), with N deposition (ammonium nitrate) applied every 3 weeks over 11 weeks for each subperiod. During each subperiod, for the first two N applications, this study stimulated 1 kg N/ha/y and 10 kg N/ha/y for ambient and increased N deposition, respectively. For the third application, 10 kg N/ha/y and 50 kg N/ha/y were applied for ambient and increased N deposition, respectively, enabling greenhouse gas measurement. Thus, each growth season received a total of 12 kg N/ha/y under ambient N deposition, and 70 kg N/ha/y under increased deposition, representing the existing and projected future levels, respectively. More detailed setup information was presented in Jia et al. (2021).

Moreover, each microcosm received 75 mL of microbial wash to correct for potential differences between different treatments and natural conditions. The microbial wash was prepared by wet-sieving 3 kg soil-inoculum mixtures (2.5 kg of fresh soil and 500 g of AM fungal inoculum) through a 10-μm sieve with 6 L of deionized water. Additionally, the microcosms were watered to the 100% water holding capacity to achieve homogenization. Seeds of the 10 different plant species were sterilized with 1.25% sodium hypochlorite for 10 min, and subsequently germinated in sterilized Petri dishes. Seedlings were approximately 1 week old when planted. After equilibration for 2 days following microbial wash addition, this study introduced 33 plant seedlings in each microcosm. Died seedlings were only replaced within the first 2 weeks, which was the initial phase of the experiment, to ensure that all microcosms contained 33 healthy plants before entering the study periods. Soil moisture was maintained between 24 and 29% (equivalent to 63–75% water-holding capacity) by top-watering daily or every other day. Greenhouse conditions were set to 16-h days with a temperature regime at 25/17 °C (day/night) and relative humidity of ~65%/85%.

### Sampling and soil physiochemical properties

At the end of each subperiod (including the last day of periods 1, 2, and 3, respectively), each plant was harvested approximately 2 cm above the soil surface, separated by species, dried at 65 °C for 72 h, and then weighed to calculate plant community biomass and Shannon–Wiener diversity. The total weight of all plants in each microcosm was defined as community biomass. Two soil cores were taken from each microcosm during periods 1 and 2, and four cores at the end of period 3. The soil cores were immediately sieved through a 2-mm sieve to mix thoroughly and collect root samples. The soil was further divided into two factors based on soil physiochemical properties analysis, such as air-dried soil for pH and Olsen-P measurement; and frozen soil (−20 °C) for measuring NH_4_^+^-N, NO_3_^−^-N, and soil DNA concentration. Soil pH was detected using a pH meter (PH211, HANNA, Italy); Olsen-P was detected with 0.5 mol/L NaHCO_3_, following the molybdate blue colorimetric method; and soil NH_4_^+^-N and NO_3_^−^-N were determined using a continuous flow analyzer (Futural II, Alliance Instruments Ltd., France). These methods were described by Agroscope (1996). Soil DNA concentration has been recognized as a valuable indicator of soil microbial biomass and was therefore used in the present study (Gong et al., 2021; Riah-Angleta et al., 2014). Soil DNA was extracted from 0.5 g of soil using the FastDNA® spin kit for soil (MP Biomedicals, Switzerland). Furthermore, AM fungal colonization parameters (such as AM fungal arbuscular colonization, AM fungal hyphae colonization, AM fungal vesicular colonization, and AM fungal total colonization) of each period were quantified. The detailed measurement method of the above parameters was presented and has been published in one of our earlier studies (Jia et al., 2021). Thus, the raw data and ANOVA analyses of these parameters are presented in the Supplementary Tables S4, S5. Notably, in our previous study, the effects of AM fungi were examined in relation to the stability of ecosystem multifunctionality under increased N deposition, but the AM fungi on soil CH_4_ and CO_2_ fluxes under increased N deposition was not explored–this being the core focus of the present study.

### Measurement of soil CH_4_ and CO_2_ fluxes

At the end of each subperiod, an N pulse was applied to enable monitoring the soil CH_4_ and CO_2_ fluxes. A removable airtight cap (inner diameter 15 cm, and a height of 10 cm above the soil surface) was used to seal the headspace of each microcosm after plant harvesting. The monitoring process followed Jia et al. (2021). Briefly, the headspace was sealed for 4 min, and the gas was directly tested using Picarro G2508 Greenhouse Gas Analyzer (Picarro Inc.). Fluxes were calculated from the linear change rates of CH_4_ (ug CH_4_-C/m^2^/h) and CO_2_ (ug CO_2_-C/m^2^/h) concentrations, using the following formula: F_gas_ = V/A × Slope×(M*101.3 × 10^3^)/(8.314*T) × 10^−6^, where V is the chamber volume (m^3^); A is the base area of chamber volume (m^2^); Slope is the linear change rate of the gas; M is the molar mass of CH_4_ or CO_2,_ and CH_4_-C is calculated using CH_4_*14/16, CO_2_-C is calculated using CO_2_*12/44; and T is the measurement temperature. Data from the first 30 s were excluded from the linear regression to minimize the potential interference from the installation process between adjacent microcosms.

First, the background level of soil CH_4_ and CO_2_ fluxes was measured for each microcosm before applying N pulse. After 1 day, the N pulse was applied to the ambient and increased N deposition, respectively, and the soil CH_4_ flux was monitored until recovering to the background level. During the first 3 days, soil CH_4_ and CO_2_ fluxes were monitored thrice per day (morning, afternoon, and night). Over the following 2 days, fluxes were monitored twice per day (morning and afternoon). Thereafter, fluxes were monitored once per day (only morning) until the fluxes recovered to the background level. Cumulative CH_4_ and CO_2_ emissions were calculated over the 7-day period for each subperiod. In total, the study monitored soil CH_4_ and CO_2_ fluxes for 7 days during per subperiod, resulting in 16 gas flux measurements per microcosm, and 512 measurements throughout the entire experimental period.

### Statistical analyses

**Figure 1:**
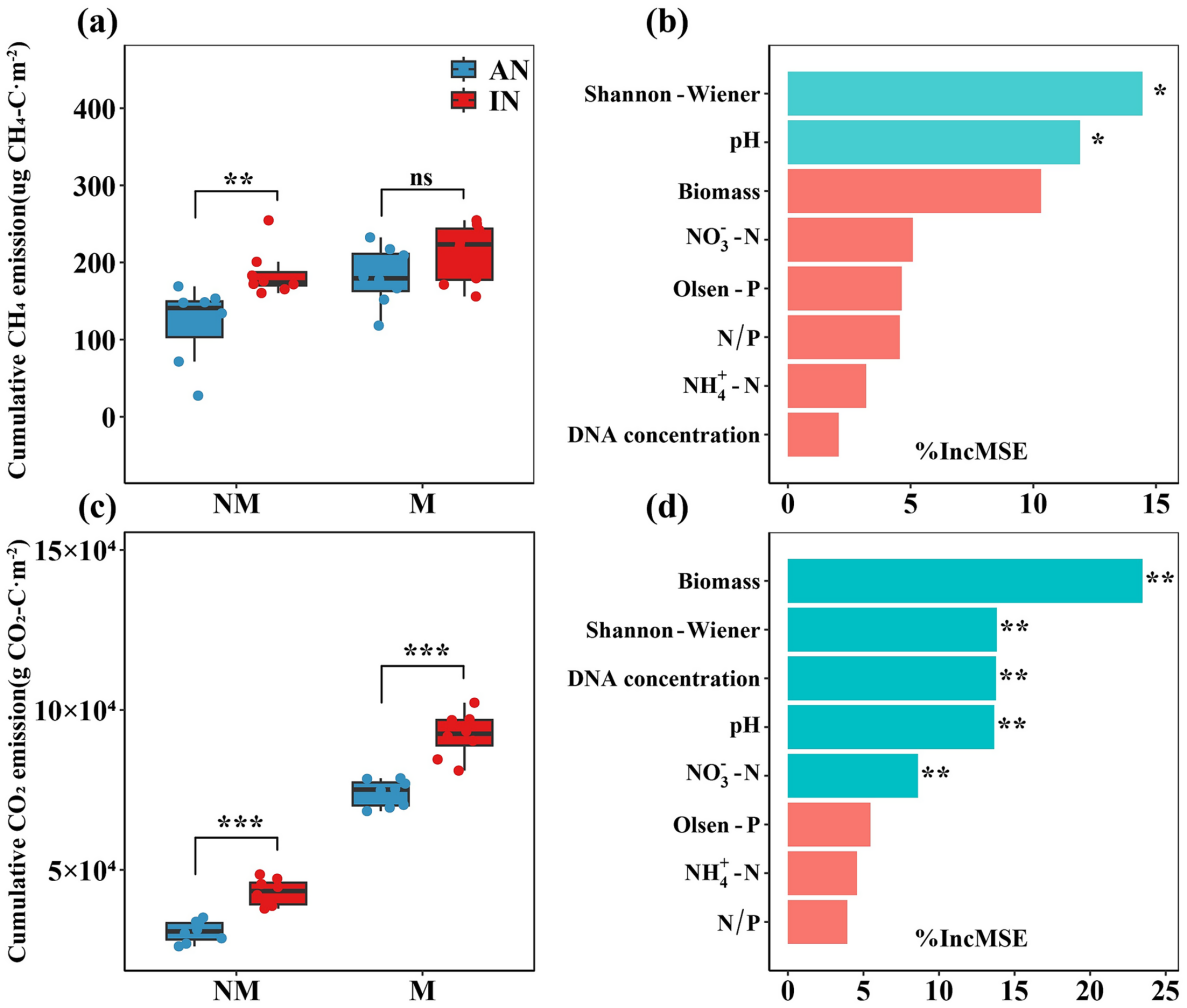
Cumulative CH_4_ and CO_2_ emission as affected by AM fungi and increased N deposition across the whole period **(a,c)**, random forest analysis determined the roles of plant community Shannon–Wiener diversity, plant community biomass, and soil parameters in contributing to the soil cumulative CH_4_ and CO_2_ emissions **(b,d)**. NM, No AM fungi treatment; M, With AM fungi treatment; AN, Ambient nitrogen deposition; IN, Increased nitrogen deposition; NO_3_^−^-N, Soil nitrite nitrogen; NH_4_^+^-N, Soil ammonium nitrogen. Cumulative CH_4_ and CO_2_ emissions during individual periods were shown in Supplementary Figure S1, and ANOVA results were shown in Supplementary Tables S2, S3. *, *p* < 0.05; **, *p* < 0.01; ***, *p* < 0.001; ns, Non-significant.

All statistical analyses were conducted in the R version 4.3.3 statistical software. At first, three-way repeated measures ANOVA was performed to test the main and interactive effects of AM fungi (AM), increased N deposition (N), and monitoring time (T) on the CH_4_ and CO_2_ fluxes during the periods 1, 2, and 3, respectively (Supplementary Table S1). Negative CH_4_ fluxes were also included in the dataset. Similarly, another three-way repeated measures ANOVA was performed to test the effects of AM, N, and Pon cumulative CH_4_ and CO_2_ emissions across the entire experimental period (Supplementary Table S2). Two-way ANOVA was further performed to evaluate the effects of AM fungi, increased N deposition, and their interaction on the cumulative CH_4_ and CO_2_ emissions during periods 1, 2, and 3, and the whole period, respectively (Supplementary Table S3). Subsequently, *t-*tests were performed to compare the differences in cumulative CH_4_ and CO_2_ emissions between ambient N deposition (AN) and increased N deposition (IN) with or without AM fungi during periods 1, 2, and 3, and the entire experimental period (Figure 1; Supplementary Figure S1). Normality and homoscedasticity of the dataset were formally tested prior to ANOVA analysis. Secondly, linear regression analyses were performed to investigate the relationships between cumulative CH_4_ and CO_2_ emissions with plant community parameters (such as Shannon–Wiener diversity and plant community biomass), soil physiochemical properties (such as soil pH, Olsen-P, inorganic N, and N/P), soil microbial biomass (such as soil DNA concentration), and AM fungal colonization parameters (such as hyphae, arbuscular vesicular, and total root colonization) under ambient N deposition, increased N deposition, and combined treatments, respectively. Notably, plant community parameters (such as Shannon–Wiener diversity and plant community biomass), soil physiochemical properties (such as soil pH, Olsen-P, inorganic N, N/P, and soil DNA concentration) have been reported by Jia et al. (2021), these parameters were further used in the present study to examine their potential effects on soil CH_4_ and CO_2_ emissions, with the raw data presented in the Supplementary Table S4. Furthermore, this study used random forest analysis based on R package “random Forest” with 500 decision trees to evaluate the relative contributions of plant community parameters (such as Shannon–Wiener diversity and plant community biomass) and soil parameters (such as soil pH, Olsen-P, NO_3_^−^-N, NH_4_^+^-N, N/P, and DNA concentration) to the cumulative CH_4_ and CO_2_ emissions. Variable importance was quantified using the percent increase in mean squared error (%IncMSE). Finally, structural equation modeling (SEM) was conducted to reveal how AM fungi and increased N deposition regulated cumulative CH_4_ emissions based on the R package “piecewise SEM.” Notably, this study first constructed a hypothetical model comprising all potential pathways of assumed causal relationships (Supplementary Figure S5). Then, updated the model and removed non-significant pathways until the model gave an adequate fit, such that, the Fisher’s C statistic was non-significant (*p* > 0.05), Akaike information criteria (AIC) did not decrease with *p*-value >0.05, and standardized path coefficients were reported to compare the relative magnitude of each effect.

## Results

**Figure 2:**
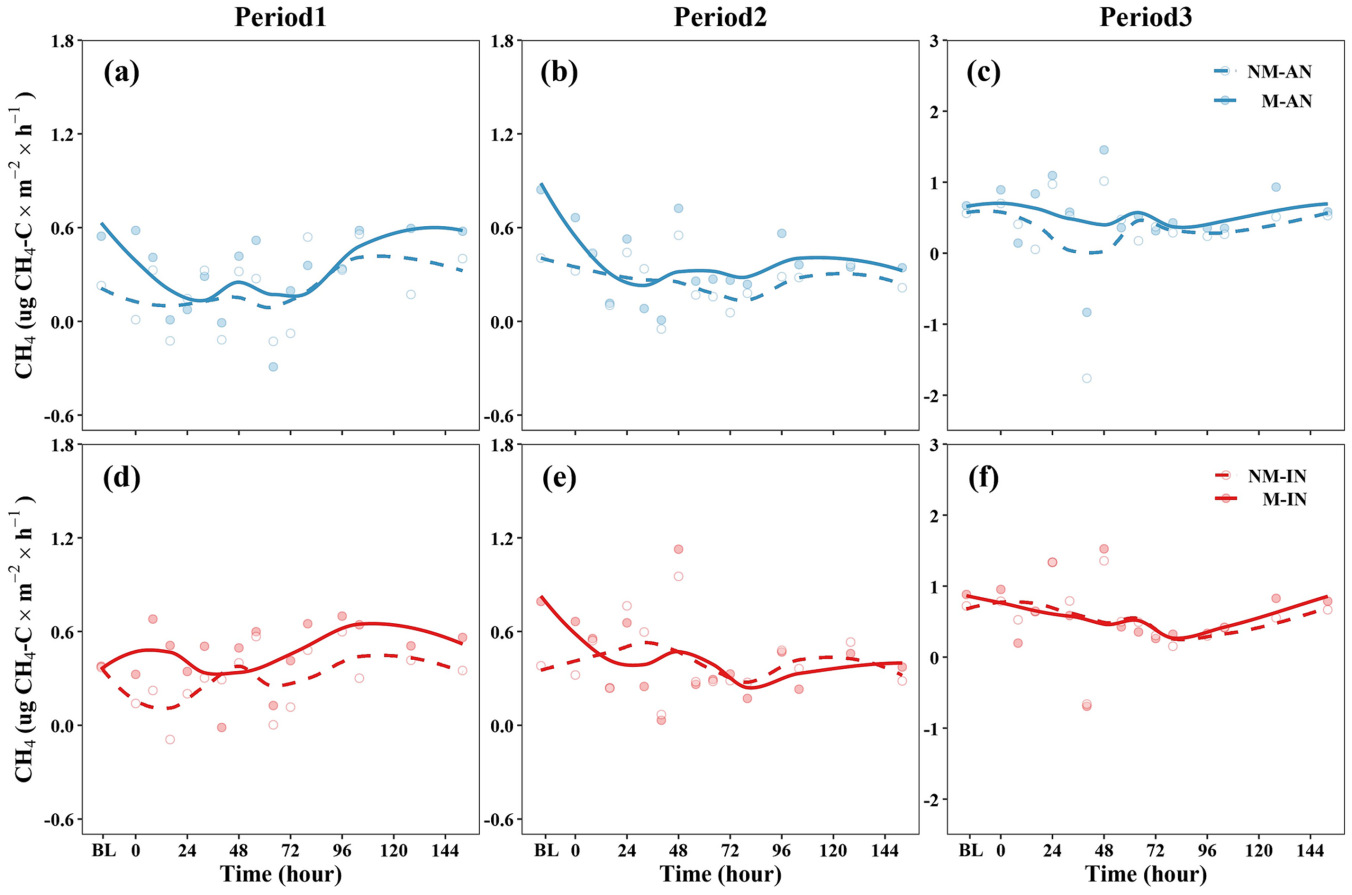
Impact of AM fungi and increased N deposition on soil CH_4_ fluxes within 7 days at the end of periods 1 **(a,d)**, 2 **(b,e)**, and 3 **(c,f)**. Hollow blue cycles with dash line represent no AM fungi with ambient N deposition treatment (NM-AN), solid blue cycles with a solid line represent AM fungi with ambient N deposition treatment (M-AN); hollow red cycles with dash line represents no AM fungi with ambient *N* deposition treatment (NM-AN), solid red cycles with solid line represents AM fungi with ambient N deposition treatment (M-AN). BL, background level. ANOVA results are depicted in Supplementary Table S1. The *y*-axis scales during period 3 are different with that in the other periods.

**Figure 3:**
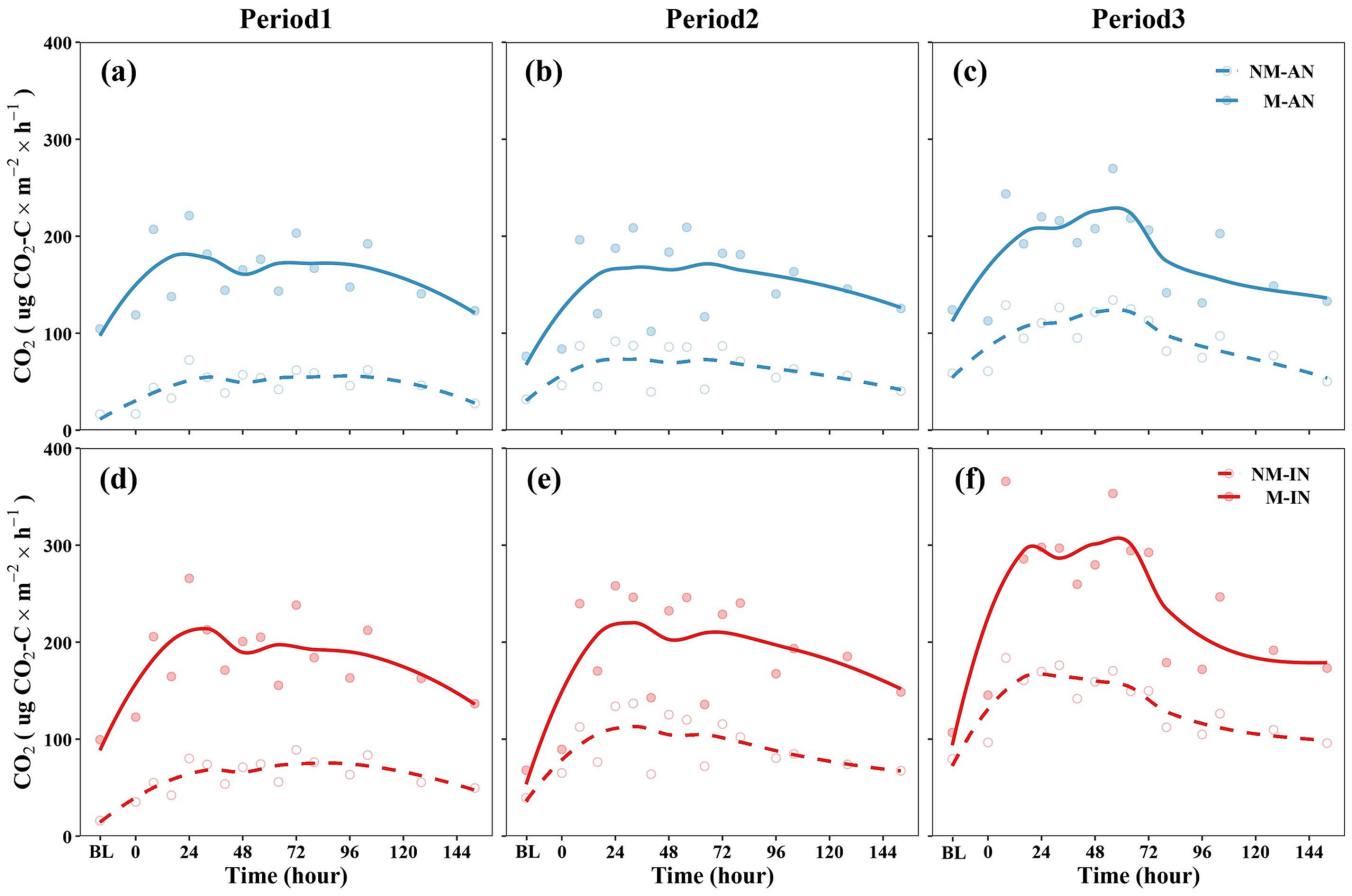
Impact of AM fungi and increased N deposition on soil CO_2_ fluxes within 7 days at the end of periods 1 **(a,d)**, 2 **(b,e)**, and 3 **(c,f)**. Hollow blue cycles with dash line represents no AM fungi with ambient N deposition treatment (NM-AN), solid blue cycles with a solid line represent AM fungi with ambient N deposition treatment (M-AN); hollow red cycles with dash line represent no AM fungi with ambient N deposition treatment (NM-AN), solid red cycles with a solid line represent AM fungi with ambient N deposition treatment (M-AN). BL, background level. ANOVA results see Supplementary Table S1.

Responses of soil CH_4_ and CO_2_ emissions to increased N deposition and AM fungi Increased N deposition significantly stimulated soil CH_4_ emissions throughout the experimental period, with an average increase of 36.7% (*p* < 0.01), but did not trigger soil CH_4_ emissions peaks after adding N pulse (Figure 2; Supplementary Table S1). This study also identified that AM fungi exerted a pronounced stimulatory effect on soil CH_4_ flux, with a significant increase of 60.6% observed only in period 1 (*p* = 0.004; Figure 2; Supplementary Table S1). For soil CO_2_ flux, the emission patterns were similar with that of soil CH_4_ flux (Figure 3). Both AM fungi and increased N deposition strongly increased soil CO_2_ flux by 124.5 and 28.5%, respectively (*p* < 0.0001; Figure 3; Supplementary Table S1). Furthermore, AM fungi and increased N deposition posed significantly interactive effects on soil CO_2_ flux across the whole period (Figure 3; Supplementary Table S1). It is worth to noting that the promoted effects of AM fungi on soil CO_2_ flux were more pronounced under increased N deposition (Figure 3).

For cumulative greenhouse gas emissions, increased N deposition and AM fungi promoted cumulative soil CH_4_ emissions during the periods 1, 2, and 3, and thus for the whole period (Figure 1; Supplementary Figure S1; Supplementary Tables S2, S3). More interestingly, effects of increased N deposition on cumulative soil CH_4_ emissions differed significantly under absence and presence of AM fungi based on the *t*-test results (Figure 1; Supplementary Figure S1). In specific, increased N deposition significantly promoted cumulative soil CH_4_ emissions by 60.7 and 78.1% in the absence of AM fungi (*p* < 0.05) during periods 2 and 3, respectively; but showed no effects on cumulative soil CH_4_ emissions in the presence of AM fungi during the periods 2 and 3 (Supplementary Figure S1). And across the whole period, these influence patterns of increased N deposition on cumulative soil CH_4_ emissions were more pronounced (Figure 1). For cumulative soil CO_2_ emissions, increased N deposition significantly enhanced cumulative soil CO_2_ emissions by 17.9, 30.6, and 36.3% during the periods 1, 2, and 3, respectively. Similarly, AM fungi also markedly increased cumulative soil CO_2_ emissions by 207.7, 125.0, and 85.8% across the three periods (*p* < 0.0001; Figure 1; Supplementary Figure S1; Supplementary Tables S2, S3). Overall, the stimulatory effect of AM fungi was considerably stronger than that of increased N deposition (Figure 1; Supplementary Figure S1).

### Underlying mechanisms of increased N deposition and AM fungi on soil CH_4_ and CO_2_ emissions

First, AM fungi significantly increased plant community biomass, Shannon–Wiener diversity, and soil DNA concentration, but reduced soil pH, Olsen-P, and soil NO_3_^−^-N across the three periods (*p* < 0.0001; Supplementary Tables S4, S5). Increased N deposition significantly increased soil NO_3_^−^-N (*p* < 0.0001), but posed limited effects on soil NH_4_^+^-N content (Supplementary Tables S4, S5). Notably, increased N deposition started to show significant effects on plant community from period 2, and also increased N deposition significantly increased plant community biomass in periods 2 and 3 (*p* < 0.001), and reduced plant community Shannon–Wiener diversity in period 3 (*p* < 0.01; Supplementary Tables S4, S5). And, both AM fungi and increased N deposition significantly increased soil N/P in period 2 (*p* < 0.05; Supplementary Tables S4, S5).

**Figure 4:**
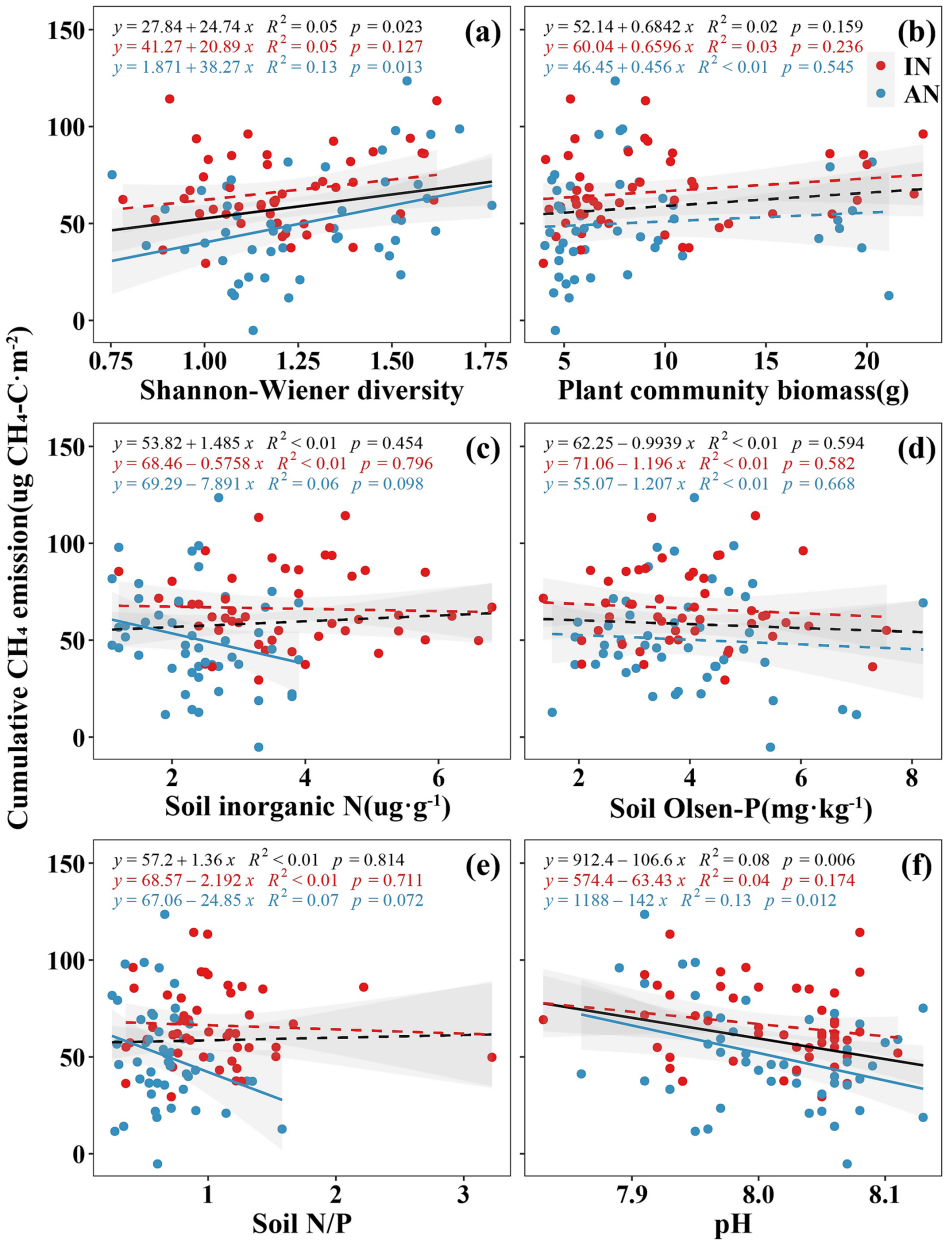
Linear regression analyses of the relationships between cumulative CH_4_ emissions with plant parameters [**(a)** plant community Shannon–Wiener diversity and **(b)** plant community biomass] and soil parameters [**(c)**, soil inorganic N; **(d)** soil Olsen-P; **(e)** soil N/P; and **(f)** soil pH]. The gray shaded area represents 95% confidence intervals. Significant linkages are shown in solid lines, and non-significant linkages are shown in dashed lines. Significant level: *p* < 0.05, notably, the *p*-value of the regression between cumulative CH_4_ emissions and soil N/P under ambient N deposition was 0.072 **(e)**.

Second, the linear regression analyses demonstrated that soil cumulative CH_4_ emissions were positively associated with plant community Shannon–Wiener diversity (*p* < 0.05; Figure 4a). Soil pH exhibited negative correlations with cumulative soil CH_4_ emissions, and this association was stronger than those of other soil parameters (*p* < 0.05; such as soil Olsen-P and soil inorganic N; Figures 4c–f). Notably, these correlative patterns were modulated by N deposition level (Figure 4). Stronger statistical associations were observed between cumulative soil CO_2_ emissions and plant community Shannon-Wiener diversity (*p* < 0.001), plant community biomass (*p* < 0.001), soil pH (*p* < 0.001), Olsen-P (*p* < 0.001), soil inorganic N (*p* < 0.001), and N/P (*p* < 0.05; Supplementary Figure S2). Moreover, both cumulative soil CH_4_ (*p* < 0.05) and CO_2_ (*p* < 0.001) emissions were positively correlated with soil DNA concentration (Supplementary Figure S3). Parameters of AM fungal colonization showed overall neutral associations with cumulative soil CH_4_ and CO_2_ emissions (Supplementary Figure S4). Specifically, AM fungal arbuscular colonization and total colonization (*p* < 0.05) were negatively and positively correlated with cumulative soil CO_2_ emissions, respectively (Supplementary Figures S4b,h). Additionally, random forest models were used to identify the key potential predictors for cumulative soil CH_4_ and CO_2_ emissions under both AM fungi and increased N deposition treatments (Figures 1b,d). Soil pH emerged as the primary soil predictor correlated with both cumulative soil CH_4_ and CO_2_ emissions (Figures 1b,d). Furthermore, it is interesting to note that the plant community Shannon–Wiener diversity was the main plant parameter predictor for cumulative soil CH_4_ emissions (Figure 1b), whereas both plant community biomass and Shannon–Wiener diversity were identified as prominent correlative predictors for cumulative soil CO_2_ emissions (Figure 1d).

**Figure 5:**
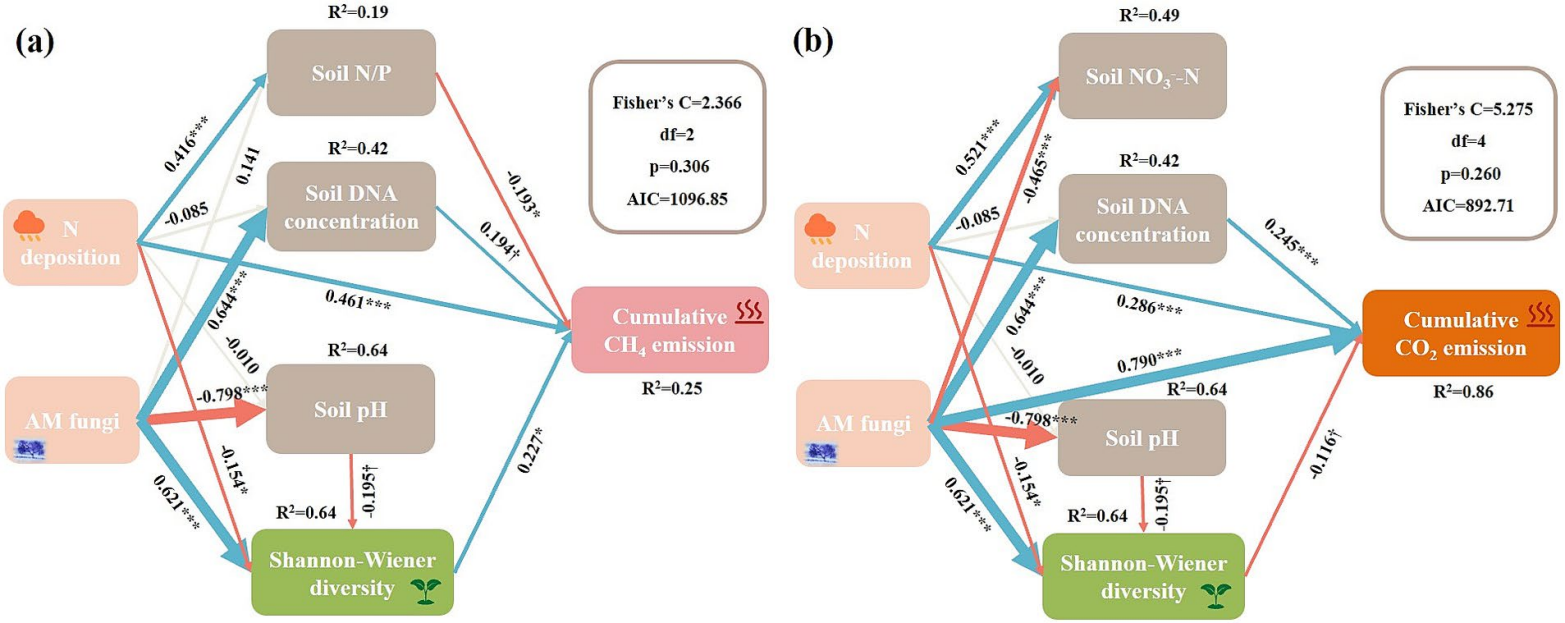
Structural equation model estimated the direct and indirect pathways of AM fungi and increased N deposition on soil cumulative CH_4_ **(a)** and CO_2_ **(b)** emissions. Standardized path coefficients are shown against arrows, where blue and red arrows indicate positive and negative pathways, respectively, at ^†^*p* < 0.1, *p < 0.05, **p < 0.01, and ***p < 0.001. Non-significant pathways are shown by gray lines.

Finally, the best-fitting SEM model further confirmed the correlative patterns derived from the random forest analysis. Plant community Shannon–Wiener diversity served as a central correlative variable that was interactively linked to AM fungi and increased N deposition in association with soil CH_4_ and CO_2_ emissions (Figure 5). Furthermore, soil pH, which was highly correlated with AM fungi, exhibited indirect statistical associations with soil CH_4_ and CO_2_ emissions via its negative correlation with plant community Shannon–Wiener diversity (Figures 5a,b). AM fungi were statistically associated with higher cumulative CH_4_ and CO_2_ emissions alongside increased soil DNA concentration, which in turn showed positive correlations with soil CH_4_ and CO_2_ emissions (Figures 5a,b). Notably, increased N deposition was directly correlated with enhanced soil CH_4_ and CO_2_ emissions (Figure 5).

## Discussion

Methane fluxes are important components of soil C flux and are significantly influenced by global climate change, such as increased N deposition perturbations (Jang et al., 2011; Wuebbles and Hayhoe, 2002; Yue et al., 2016). Involvement of AM fungi in the soil CH_4_ emissions in the face of ongoing climate change remains unclear. This limits the ability to understand the impacts of global climate change on soil CH_4_ flux. This study may be considered as a pilot study to explore the interactive effects of AM fungi and increased N deposition on soil CH_4_ emission in grassland ecosystems. With the continuous monitoring of soil CH_4_ flux spanning three growth seasons, this study reports two notable findings. First, increased N deposition stimulated soil CH_4_ emissions, but this effect was only detected in the absence of AM fungi. Second, AM fungi mitigated the stimulatory effects of increased N deposition under high N deposition perturbations, but AM fungi exerted positive effects on soil CH_4_ emissions under low N deposition–a pattern that coincided with higher plant community diversity. Overall, these findings provide empirical evidence for understanding the impacts of AM fungi on soil CH_4_ flux in the context of increased N deposition and are helpful for advancing terrestrial ecosystems carbon sequestration.

### Impacts of increased N deposition on soil CH_4_ emissions

Increased N deposition posed significant stimulatory effects on soil CH_4_ emissions across the whole period, supporting the first hypothesis and was consistent with numerous field-based observations in grasslands (Jang et al., 2011; Liu and Greaver, 2009; Yue et al., 2016). Soil N availability has been widely recognized in field experiments as a key regulator of soil CH_4_ production and oxidation processes (Cardoso et al., 2017; Cen et al., 2024; Liu and Greaver, 2009). In terms of soil N conditions, nitrogen deposition exerts a nonlinear effect on CH_4_ emissions in grasslands, exhibiting a “low inhibition high stimulation” trend (Zhang et al., 2023). For instance, a 10-year field experiment in a semi-arid meadow steppe in northeast China found that high N deposition (such as 100 kg/ha/y) promoted soil CH_4_ net emissions, mirroring the stimulatory effects (Gao et al., 2023). Similarly, a temperate desert steppe field study demonstrated that increased N deposition consistently suppressed CH_4_ oxidation and increased net CH_4_ release, aligning with the present observations (Cheng et al., 2022). Previous study with the same system indicated that soil N was in surplus during period 1 due to the autoclaved process, but soil N/P was balanced for plant growth during period 3 (Jia et al., 2023). Thus, the observed stimulation of CH_4_ under increased N deposition possibly arises from intensified competition between methane-oxidizing bacteria for NH_4_^+^-N and CH_4_, thereby reducing CH_4_ oxidation and increasing net emissions (Bodelier and Laanbroek, 2004; Peng et al., 2019). However, this proposed mechanism lacks direct experimental evidence in the present study, as key functional microbial groups (such as methanotrophs and methanogens) and their related functional genes were not measured, which limits the robustness of this inference. These results are, however, in line with the observations from alpine meadow and desert grasslands, where increased N deposition promoted soil CH_4_ emissions through the alteration of soil N availability (Yue et al., 2019; Yue et al., 2016). Notably, random forest analysis identified soil pH as the most influential soil physicochemical property impacting soil CH_4_ emissions, which was also revealed in a recent study (Tang et al., 2024). However, in contrast to part of the first hypothesis, this study detected no indirect pathway from increased N deposition to CH_4_ emissions through N-induced soil acidification. This discrepancy likely reflects the relatively short duration of N deposition in this study, whereas significant soil acidification typically develops only under long-term N deposition scenarios (Stevens, 2016). Therefore, prolonged field-based monitoring, coupled with the determination of key functional microbial groups, is needed to distinguish the various contributions of available soil N and other physicochemical parameters to soil CH_4_ emissions under chronic N deposition.

Furthermore, this study found that increased N deposition decreased soil CH_4_ emissions, which was associated with lower plant community diversity. Bulk analyses of the effects of increased N deposition on soil CH_4_ emissions, regardless of plant community, cannot fully reveal the underlying influencing mechanisms of increased N deposition on soil CH_4_ emissions (Jang et al., 2011; Yue et al., 2016). Previous studies have indicated that living plants themselves can emit CH_4_ under toxic conditions (Bruhn et al., 2012; Keppler et al., 2006). Aerenchymous tissues can also directly transport CH_4_ from soil to the atmosphere without oxidation processes, such as rice plants (Garnet et al., 2005). Additionally, higher plant community diversity is often associated with greater soil microbial community diversity and activity in field conditions, potentially increasing soil CH_4_ emissions (Harpole and Tilman, 2007). A field study in a northeast China meadow steppe further validated this linkage, showing that N-induced declines in plant diversity reduced CH_4_ emissions by lowering plant-mediated CH_4_ transport and microbial activity (Ren et al., 2019). The random forest analysis further confirmed plant community diversity as the strongest predictor of soil CH_4_ emissions. Collectively, these results emphasize that both plant community (such as diversity) and soil physicochemical properties jointly regulate soil CH_4_ emissions, consistent with holistic perspectives emerging from multi-site field studies of N deposition. Future research should explicitly address the indirect effects of increased N deposition on soil CH_4_ emissions mediated by changes in plant community, especially under realistic field N deposition scenarios.

### AM fungi regulated the effects of increased N deposition on soil CH_4_ emissions

What is most intriguing about these findings is that AM fungi reduced the stimulative effects of increased N deposition on soil CH_4_ emissions across several plant growth seasons. This study found that the inhibitory effects of AM fungi on soil CH_4_ emissions were highly dependent on the N deposition level, that is, AM fungi reduced soil CH_4_ emissions, with this effect being particularly pronounced under high N deposition. This may be associated with N transformations involved in AM fungi. There is limited published data available to compare with the observations on the effects of AM fungi on soil CH_4_ emissions, especially in the context of global climate change. One previous study conducted in rice paddies found that AM fungi significantly reduced soil CH_4_ flux by increasing the soil C:N ratio (Zhang et al., 2017). Additionally, a recent study further revealed that the alteration of soil NO_3_^−^-N was a potential pathway influencing AM fungi on soil CH_4_ emissions in desert ecosystems (Yue et al., 2020). Both of these results indicate that AM fungi may be associated with the soil CH_4_ emission processes by altering soil available N content. AM fungi are widely recognized as key players in soil C, N, and P cycles (Martin et al., 2017). Previous studies have indicated that changes in soil nitrogen availability could be associated with alterations in the community structure of methane-oxidizing bacteria and methane-producing archaea (Kollah et al., 2023; Peng et al., 2019). Thus, this study hypothesize that the changes in soil NO_3_^−^-N and NH_4_^+^-N induced by AM fungi and increased N deposition might be associated with enhanced activities of methane-oxidizing bacteria, thereby reducing soil CH_4_ emissions. However, this hypothesis needs to be confirmed by experimental evidence in the future. Additionally, based on the SEM results, the interactive effects of AM fungi and increased N deposition on soil CH_4_ emissions were mainly associated with changes in plant community Shannon–Wiener diversity. Both AM fungi and increased N deposition have been widely proven to be the major drivers of plant community diversity (Bai et al., 2010; Martin and van der Heijden, 2024), it is not surprising that plant community diversity was the central factor associated with AM fungi and increased N deposition that may influence soil CH_4_ emissions. These results highlight that plant community diversity also plays important, albeit indirect, roles in controlling CH₄ emissions, which should not be overlooked in future studies.

Furthermore, it is interesting to note that AM fungi promoted soil CH_4_ emissions under low N deposition. Two potential reasons may explain the AM fungal stimulatory effects. First, soil pH was a major factor associated with soil CH_4_ emissions based on the random forest analysis, and the SEM model showed that AM fungi significantly reduced soil pH. AM fungi are widely reported to decrease soil pH through multiple pathways, such as exudation of organic acids (such as citrate, malate, and oxalate), and enhancement of proton extrusion during nutrient uptake (such as NH_4_^+^-N; Martin and van der Heijden, 2024; van der Heijden et al., 2015). Moreover, AM fungi can intensify rhizosphere acidification indirectly by promoting plant growth and root exudation, as diverse plant communities diversity release organic acids and secondary metabolites that could further lower rhizosphere pH (Philippot et al., 2013; van der Heijden et al., 2015). Soil microbes growing in high pH conditions are sensitive to soil acidification (Zhang et al., 2024); the decline in soil pH induced by AM fungi could be associated with enhanced soil microbial activities, which in turn, promoted soil CH_4_ emissions (Täumer et al., 2021). The soils used in this experiment were alkaline with an average soil pH of 8.1. The linear regressions revealed that soil CO_2_ emissions increased with soil pH reduction, indicating higher soil microbial activity at lower soil pH conditions. The clear increase in soil CH_4_ emissions with reducing soil pH further supports this potential explanation. Second, the promotion of soil CH_4_ emissions by AM fungi was mainly associated with increased plant community diversity, as revealed by our SEM analysis. The increases in plant community diversity could be associated with enhanced soil microbial activity and diversity due to their tight relationships (Wardle et al., 2004), which were also indicated by the increased CO_2_ emissions in the presence of AM fungi. These results clearly documented that soil DNA content was positively related to the soil CH_4_ emissions. Notably, these results further revealed that soil CO_2_ emissions were more strongly promoted by AM fungi under increased N deposition compared to low N deposition; however, soil CH_4_ emissions were not promoted by AM fungi under increased N deposition. These different responses in soil CO_2_ and CH_4_ emissions to AM fungi suggest that AM fungi may stimulate soil microbial activities to mineralize nutrients, rather than for the growth of microbes themselves. Furthermore, it is commonly accepted that soil CH_4_ emission processes occur mainly through non-AM fungi (Jiang et al., 2025; Liu and Greaver, 2009). While this study did not directly detect the changes in soil microbial community (such as methanotrophic bacteria and methanogenic archaea) induced by AM fungi. Thus, the process and potential influence mechanisms by which AM fungi are associated with the soil microbial community require further investigation in future studies to establish a more unambiguous and generalizable influence pattern.

### Limitations and implications for future research

In summary, this study reveals the stimulative effects of increased N deposition on soil CH_4_ emissions, highlighting the importance of AM fungi in mitigating soil CH_4_ emissions under the background of global climate change in grassland ecosystems. While the study provides valuable insights into the role of AM fungi in mitigating increased N deposition-induced CH_4_ emissions, notable limitations existing in the present study. One key limitation is that the results were obtained using an artificial plant community and soil. In nature, soil microbial abundance and biodiversity are high, with complex interaction networks and functional complexity driving various nutrient cycling processes. Furthermore, natural environmental conditions, such as temperature and soil moisture, are unpredictable and change rapidly, yet they play dominant roles in soil CH_4_ emissions (D'Imperio et al., 2017; White et al., 2023). Additionally, soils sterilized by the autoclaved method could significantly increase soil organic matter content and the activity of pathogenic bacteria, as well as damage soil structures (such as aggregate structure; Perkins et al., 2013). Considering this, the approach of using an artificial system with a relatively homogenous soil environment may have underestimated the importance of complex soil microbial processes and their roles in affecting soil CH_4_ emissions (Wagg et al., 2019). Meanwhile, the focus on soil functional microbe-AM fungi represents only a fraction of the diverse soil biota. A limitation relevant to the central claim of this study is that it did not directly determine the responses of methanogenic archaeal and methanotropic bacterial communities to increased N deposition and AM fungi, which are directly responsible for CH_4_ production and oxidation processes, respectively (Le Mer and Roger, 2001; White et al., 2023). Thus, study cannot explicitly confirm whether AM fungi selectively promote methanogens, suppress methanotrophs, or alter their ratio, which may be essential for fully elucidating the mechanisms by which AM fungi regulate soil CH_4_ emissions. This might explain the relatively low explained variance in soil CH_4_ emissions (25%) from the SEM model. To the best our knowledge, no studies are focusing on this process so far. Considering this, microbial community analysis would be critical to answering this question, and the next challenge is to link the responses of soil microbes, especially methanogenic archaea and methanotrophic bacteria, to clarify the roles of AM fungi in regulating soil CH_4_ emissions.

Additionally, these results showed that net CH_4_ flux in the system indicated the experimental soils acted as a net CH_4_ source, which is inconsistent with the common understanding that grassland soils act as sinks for atmospheric CH_4_ (Le Mer and Roger, 2001; Wang X. et al., 2025). The reason for this shift from a CH_4_ sink to a source is likely multifaceted. Soil autoclaving is known to profoundly alter soil physicochemical properties and reshape the inherent soil microbial community structure, which inevitably impairs the abundance and activity of methanotrophs responsible for soil CH_4_ oxidation (Wagg et al., 2019). Although microbial wash was applied to recover the sterilized soil microbiome, this compensation effect was limited and insufficient to fully restore the original soil community composition, including the metabolic capacity of methanotrophic communities. Consequently, the soil methane oxidation potential was markedly suppressed, while methanogenic processes remained relatively less constrained, ultimately driving the conversion of grassland soil from a CH_4_ sink to a net CH_4_ source. Furthermore, autoclaving severely disrupts soil physical structure and drastically reduces overall soil microbial diversity and functional complexity (Wagg et al., 2019). Such profound soil microbial community degradation cannot be fully offset by subsequent addition of microbial wash. The resultant loss of microbial functional integrity weakens soil nutrient cycling and the coordination of above- and below-ground ecological processes. Collectively, these sterilization-induced legacy effects substantially altered baseline soil CH₄ cycling capacity, which should be explicitly considered when interpreting the results and extrapolating the findings to natural field grassland ecosystems. Meanwhile, long-term increased N deposition strongly reduces soil microbial diversity and simplifies interactions among soil microbes (Bai et al., 2010; Treseder, 2008). Thus, these results indicate that the CH_4_ sink capacity of grassland ecosystems might be weakened or even switch to a source under increased N deposition in the future, especially under conditions of soil disturbance that disrupts soil microbial community structure (Zhang et al., 2020). Furthermore, soil respiration (such as CO_2_ emissions) was significantly promoted by AM fungi. Previous studies have also revealed that AM fungi can increase soil respiration by enhancing plant community biomass and soil microbial activity (Paterson et al., 2016; Yue et al., 2020). Presently, soil C accumulation through AM fungal pathways is a research hotspot in the context of global warming (Hawkins et al., 2023; Horsch et al., 2023). However, most of these studies focus primarily on soil C formation, and the effects of AM fungi on soil CH_4_ and CO_2_ emissions are largely overlooked (Hawkins et al., 2023). Future research should consider various soil C cycling processes to quantify the AM fungal effects on carbon cycles, including explicit measurements of methanogenic and methanotrophic communities, and assess the roles of AM fungi in soil net carbon accumulation. Finally, the present research is far from sufficient to clearly reveal the underlying mechanisms by which AM fungi are involved in the complex process of CH_4_ emissions. Nevertheless, this study, as a pilot study, may open a new avenue in current soil carbon accumulation research regarding the roles of AM fungi and their ecological implications, with future work focusing on soil microbial community dynamics to address the key knowledge gaps identified here.

## Conclusion

The study hypotheses found that increased N deposition promoted soil CH_4_ emissions, but this effect was detected only in the absence of AM fungi. AM fungi exhibited distinct associative effects on soil CH_4_ emissions under different N levels, with these patterns potentially linked to their role in modulating soil nutrient cycling and plant community diversity. Specifically, under low N deposition, AM fungi enhanced soil CH_4_ emissions, which coincided with higher plant community diversity; in contrast, AM fungi significantly mitigated the stimulatory effects of increased N deposition on soil CH_4_ emissions. Furthermore, plant community diversity based on Shannon–Wiener index emerged as a key associated factor, which was interactively linked to increased N deposition and AM fungi in statistical association with soil CH_4_ emissions. These findings are importance for predicting how AM fungi modulate soil CH_4_ emissions under changing global scenarios, and provide novel insights to the functional roles of AM fungi within the global carbon cycle.

## Data Availability

The original contributions presented in the study are included in the article/Supplementary material, further inquiries can be directed to the corresponding author.
