# Supplementary material for: Symbiotic soil fungi mitigate nitrogen-driven methane emissions in an experimental grassland
Source: Front Microbiol. 2026 Jun 10;17:1815239. doi: 10.3389/fmicb.2026.1815239 (PMC13290945; doi:10.3389/fmicb.2026.1815239)
Supplement: Supplementary file 1 [file Data_Sheet_1.docx]

**TABLE S1** Results of repeated measures ANOVA on the effects of different monitoring time point (T), AM fungi (AM), increased N deposition (N) and their interactions on CH_4_ and CO_2_ flux during period 1, 2, and 3, respectively. *P* values in bold are significantly different (*p*<0.10).

| **Source of variation** | | | | | | | | | | | | | | |
| --- | --- | --- | --- | --- | --- | --- | --- | --- | --- | --- | --- | --- | --- | --- |
|  | AM (*df*=1) | | N (*df*=1) | | T (*df*=15) | | AM*N (*df*=1) | | AM*T (*df*=1) | | N*T (*df*=1) | | AM*N*T (*df*=1) | |
|  | F | *p* | F | *p* | F | *p* | F | *p* | F | *p* | F | *p* | F | *p* |
| **CH_4_** |  |  |  |  |  |  |  |  |  |  |  |  |  |  |
| Period 1 | 17.12 | **0.004** | 12.17 | **0.01** | 4.34 | **0.004** | 0.31 | 0.60 | 0.84 | 0.49 | 1.14 | 0.36 | 1.35 | 0.27 |
| Period 2 | 3.07 | 0.12 | 28.40 | **0.001** | 9.95 | **<0.001** | 2.71 | 0.14 | 3.34 | **0.027** | 3.38 | **0.011** | 0.57 | 0.71 |
| Period 3 | 1.04 | 0.34 | 14.29 | **0.007** | 4.25 | **0.022** | 5.55 | **0.051** | 0.34 | 0.66 | 1.57 | 0.24 | 0.81 | 0.44 |
| **CO_2_** |  |  |  |  |  |  |  |  |  |  |  |  |  |  |
| Period 1 | 695.0 | **<0.001** | 14.37 | **0.007** | 41.15 | **<0.001** | 0.44 | 0.53 | 16.51 | **<0.001** | 3.76 | **0.013** | 2.65 | **0.056** |
| Period 2 | 506.6 | **<0.001** | 339.7 | **<0.001** | 52.37 | **<0.001** | 24.67 | **0.002** | 26.18 | **<0.001** | 7.32 | **0.001** | 2.08 | 0.125 |
| Period 3 | 556.6 | **<0.001** | 114.0 | **<0.001** | 61.16 | **<0.001** | 26.31 | **0.001** | 9.59 | **<0.001** | 6.93 | **0.001** | 2.25 | 0.101 |

**TABLE S2** Results of repeated measures ANOVA on the effects of AM fungi (AM), increased N deposition (N), experimental period (P), and their interactions on cumulative CH_4_ and CO_2_ emission, respectively. *P* values in bold are significantly different (*p*<0.10).

| **Source of variation** | | | | | | | | | | | | | | |
| --- | --- | --- | --- | --- | --- | --- | --- | --- | --- | --- | --- | --- | --- | --- |
|  | AM (*df*=1) | | N (*df*=1) | | P (*df*=15) | | AM*N (*df*=1) | | AM*P (*df*=1) | | N*P (*df*=1) | | AM*N*P (*df*=1) | |
|  | F | *p* | F | *p* | F | *p* | F | *p* | F | *p* | F | *p* | F | *p* |
| **CH_4_** | 5.267 | **0.055** | 21.889 | **0.002** | 20.789 | **0.002** | 2.046 | 0.196 | 4.327 | **0.069** | 0.05 | 0.952 | 2.429 | 0.169 |
| **CO_2_** | 1027.0 | **<0.0001** | 1549.0 | **<0.0001** | 1388.0 | **<0.0001** | 13.749 | **0.008** | 7.783 | **0.022** | 12.027 | **0.008** | 3.941 | **0.081** |

**TABLE S3** Results of two-way factorial ANOVA on the effects of AM fungi (AM), increased N deposition (N) and their interactions on cumulative CH_4_ and CO_2_ emission during period 1, 2, 3, and the whole period. *P* values in bold are significantly different (*p*<0.10).

|  | **Source of variation** | | | | | | |
| --- | --- | --- | --- | --- | --- | --- | --- |
|  |  | AM (*df*=1) | | N (*df*=1) | | AM*N (*df*=1) | |
|  |  | F | *p* | F | *p* | F | *p* |
| CH_4_ | Period 1 | 12.25 | **0.002** | 7.90 | **0.009** | 0.55 | 0.46 |
|  | Period 2 | 0.49 | 0.49 | 9.41 | **0.005** | 2.47 | 0.13 |
|  | Period 3 | 4.37 | **0.046** | 2.69 | 0.112 | 2.31 | 0.14 |
|  | Total | 10.17 | **0.004** | 11.93 | **0.002** | 1.53 | 0.23 |
| CO_2_ | Period 1 | 606.6 | **<0.001** | 15.78 | **<0.001** | 0.40 | 0.53 |
|  | Period 2 | 722.2 | **<0.001** | 86.04 | **<0.001** | 2.02 | 0.17 |
|  | Period 3 | 298.2 | **<0.001** | 78.28 | **<0.001** | 3.89 | **0.059** |
|  | Total | 744.2 | **<0.001** | 80.64 | **<0.001** | 2.86 | **0.10** |

**TABLE S4** Raw data of plant community and soil parameters which were used in the linear regression, random forest, and structural equation model analysis. Plant community parameters: plant community biomass and Shannon-Wiener diversity. Soil parameters: soil pH, Olsen-P, NO_3_^-^-N, NH_4_^+^-N, N/P, and soil DNA concentration. Data was shown in mean ± SE.

| **Period** | **treatment** | **Plant community biomass (g/pot)** | **Plant community Shannon-Wiener diversity** | **pH** | **Olsen-P (mg/kg)** | **NO_3_ˉ-N (mg/kg)** | **NH_4_+-N (mg/kg)** | **Soil N/P** | **Soil DNA concentration (ng/g)** |
| --- | --- | --- | --- | --- | --- | --- | --- | --- | --- |
| Period 1 | NM-AN | 5.22±0.19b | 1.09±0.03b | 8.06±0.01a | 5.25±0.55a | 1.98±0.19b | 1.21±0.28a | 0.66±0.09b | 324.87±50c |
|  | M-AN | 19.17±0.41a | 1.30±0.05a | 7.99±0.01b | 3.03±0.48b | 0.34±0.07c | 1.25±0.23a | 0.68±0.18b | 3199.2±339a |
|  | NM-IN | 5.02±0.26b | 1.08±0.04b | 8.06±0.00a | 5.49±0.48a | 2.58±0.31a | 1.14±0.29a | 0.75±0.14ab | 725.06±108b |
|  | M-IN | 19.53±0.84a | 1.27±0.06a | 7.99±0.01b | 3.53±0.49b | 1.59±0.27b | 1.45±0.25a | 0.99±0.22a | 3686.3±280a |
| Period 2 | NM-AN | 5.63±0.23c | 1.13±0.04c | 8.05±0.02a | 3.78±0.51a | 0.45±0.19b | 1.89±0.12a | 0.66±0.08c | 1796.4±361b |
|  | M-AN | 9.78±0.28b | 1.52±0.04a | 7.95±0.02b | 2.73±0.18b | 0.09±0.05c | 2.11±0.19a | 0.82±0.08bc | 3189.4±432a |
|  | NM-IN | 6.25±0.39c | 1.11±0.02c | 8.05±0.01a | 3.99±0.28a | 1.61±0.30a | 2.06±0.14a | 0.93±0.09b | 522.7±70.2c |
|  | M-IN | 11.43±0.35a | 1.38±0.05b | 7.96±0.02b | 2.44±0.21b | 1.18±0.52ab | 2.24±0.11a | 1.44±0.26a | 3722.4±540a |
| Period 3 | NM-AN | 4.49±0.08d | 0.99±0.05c | 8.08±0.01a | 4.68±0.22a | 0.93±0.18b | 2.00±0.17a | 0.63±0.05b | 3926.1±475a |
|  | M-AN | 7.70±0.16b | 1.57±0.02a | 7.94±0.01b | 3.73±0.21b | 0.15±0.07c | 1.98±0.21a | 0.58±0.06b | 4698.6±403a |
|  | NM-IN | 5.74±0.18c | 0.94±0.03c | 8.07±0.01a | 4.78±0.22a | 2.89±0.25a | 2.28±0.17a | 1.10±0.11a | 2138.1±396b |
|  | M-IN | 9.09±0.26a | 1.42±0.04b | 7.94±0.01b | 3.54±0.20b | 0.96±0.11b | 2.38±0.20a | 0.95±0.06a | 4543.5±347a |

**TABLE S5** Results of two-way factorial ANOVA on the effects of AM fungi (AM), increased N deposition (N) and their interactions on soil pH, Olsen-P, soil NO_3_^-^-N, NH_4_^+^-N, soil N/P, and soil DNA concentration during period 1, 2, 3, respectively. *P* values in bold are significantly different (*p*<0.05).

|  | **Source of variation** | | | | | | |
| --- | --- | --- | --- | --- | --- | --- | --- |
|  |  | AM (*df*=1) | | N (*df*=1) | | AM*N (*df*=1) | |
|  |  | F | *p* | F | *p* | F | *p* |
| **Period 1** | Plant community biomass | 822.8 | **<0.0001** | 0.03 | 0.87 | 0.32 | 0.58 |
|  | Plant community Shannon-Wiener | 17.6 | **<0.0001** | 0.33 | 0.57 | 0.04 | 0.85 |
|  | pH | 51.9 | **<0.0001** | 0.02 | 0.90 | 0.58 | 0.46 |
|  | Olsen-P | 17.3 | **<0.0001** | 0.54 | 0.47 | 0.07 | 0.80 |
|  | Soil NO_3_^-^ | 32.3 | **<0.0001** | 16.1 | **<0.0001** | 1.98 | 0.17 |
|  | Soil NH_4_^+^ | 0.44 | 0.51 | 0.06 | 0.82 | 0.27 | 0.61 |
|  | Soil N/P | 0.61 | 0.44 | 1.50 | 0.23 | 0.42 | 0.52 |
|  | Soil DNA concentration | 164.2 | **<0.0001** | 3.80 | 0.061 | 0.04 | 0.85 |
| **Period 2** | Plant community biomass | 215.5 | **<0.0001** | 12.7 | **0.001** | 2.63 | 0.12 |
|  | Plant community Shannon-Wiener | 65.8 | **<0.0001** | 3.90 | 0.058 | 2.45 | 0.13 |
|  | pH | 31.7 | **<0.0001** | 0.11 | 0.74 | 0.01 | 0.91 |
|  | Olsen-P | 22.4 | **<0.0001** | 0.61 | 0.44 | 4.19 | **0.05** |
|  | Soil NO_3_^-^ | 1.61 | 0.22 | 12.7 | **<0.001** | 0.01 | 0.91 |
|  | Soil NH_4_^+^ | 1.88 | 0.18 | 1.06 | 0.31 | 0.03 | 0.87 |
|  | Soil N/P | 5.02 | **0.03** | 9.07 | **0.005** | 1.40 | 0.25 |
|  | Soil DNA concentration | 34.4 | **<0.0001** | 0.89 | 0.35 | 5.32 | **0.029** |
| **Period 3** | Plant community biomass | 328.6 | **<0.0001** | 53.2 | **<0.0001** | 0.14 | 0.71 |
|  | Plant community Shannon-Wiener | 212.5 | **<0.0001** | 7.58 | **<0.01** | 1.48 | 0.23 |
|  | pH | 183.8 | **<0.0001** | 1.13 | 0.30 | 0.19 | 0.67 |
|  | Olsen-P | 25.9 | **<0.0001** | 0.01 | 0.93 | 0.31 | 0.58 |
|  | Soil NO_3_^-^ | 65.8 | **<0.0001** | 69.5 | **<0.0001** | 11.9 | **0.002** |
|  | Soil NH_4_^+^ | 0.04 | 0.84 | 3.29 | 0.08 | 0.11 | 0.74 |
|  | Soil N/P | 1.82 | 0.19 | 33.5 | **<0.0001** | 0.42 | 0.52 |
|  | Soil DNA concentration | 15.2 | **<0.001** | 5.68 | **0.024** | 4.01 | 0.055 |


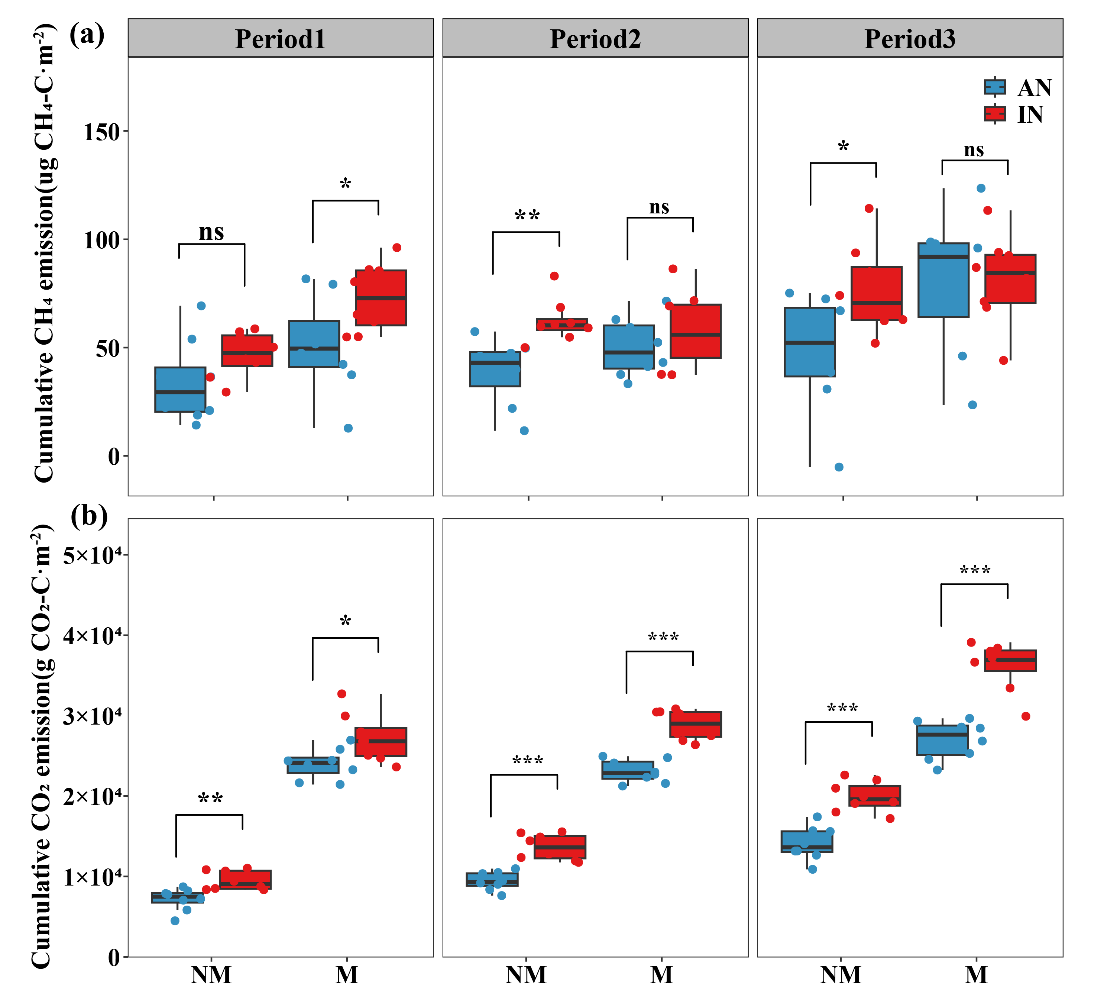


**FIGURE S1** **Cumulative CH_4_ and CO_2_ emission as affected by AM fungi and increased N deposition during period 1, 2, and 3, respectively.** NM, no AM fungi treatment; M, with AM fungi treatment; AN, ambient nitrogen deposition; IN, increased nitrogen deposition. ANOVA results see Table S2 and S3. *, p<0.05; **, p<0.01; ***, p<0.001; ns, non-significant.


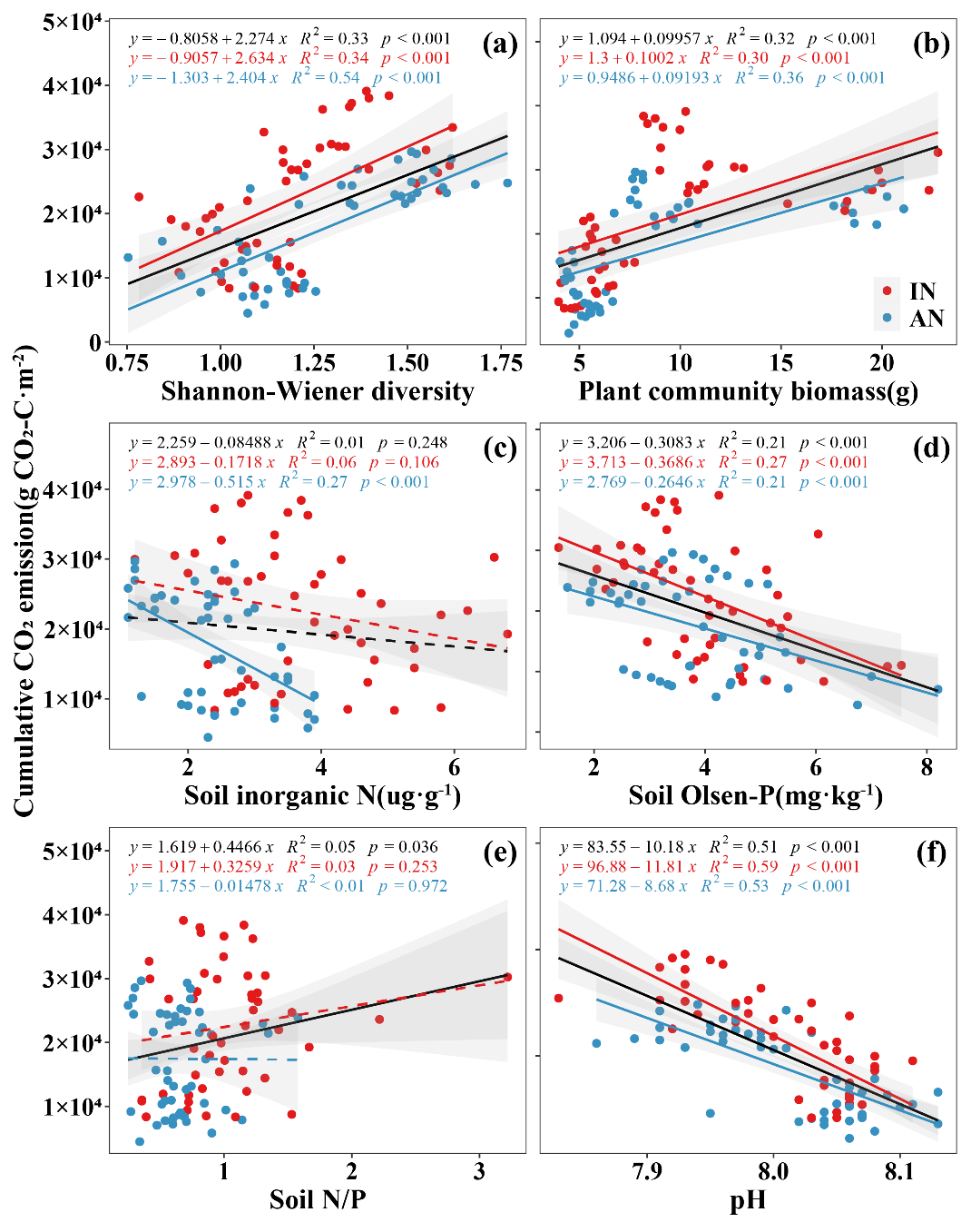


**FIGURE S2 Linear regression analyses of the relationships between cumulative CO_2_ emissions with plant parameters (a, plant community Shannon-Wiener diversity; b, plant community biomass) and soil parameters (c, soil inorganic N; d, soil Olsen-P; e, soil N/P; f, soil pH).** The gray shaded area represents 95% confidence intervals. Significant level: p<0.05.

**
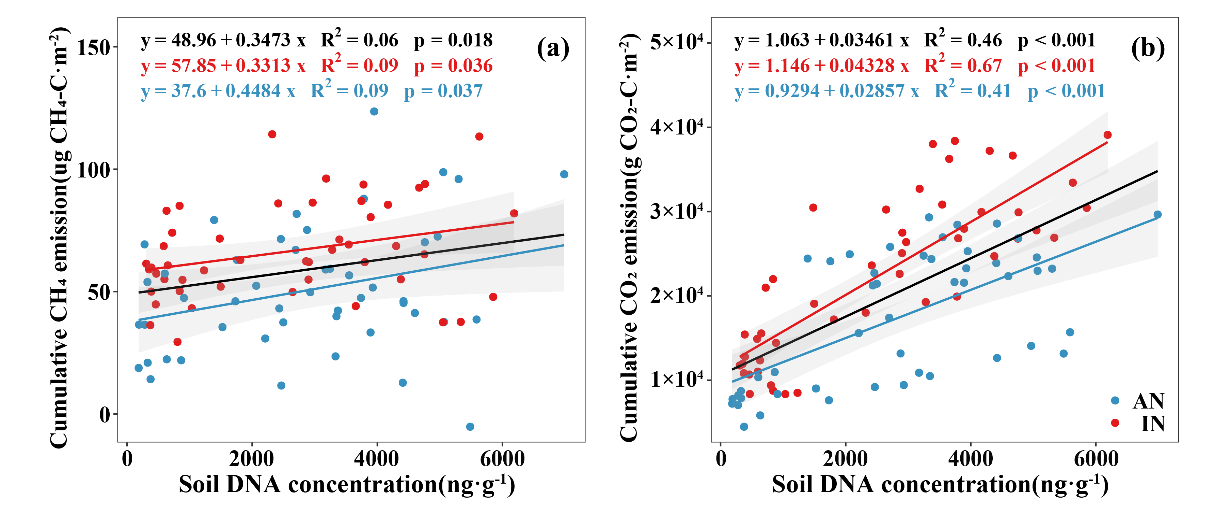
**

**FIGURE S3 Linear regression analyses of the relationships between soil DNA concentration with cumulative CH_4_ emissions (a) and cumulative CO_2_ emissions (b).** The gray shaded area represents 95% confidence intervals. Significant level: p<0.05.


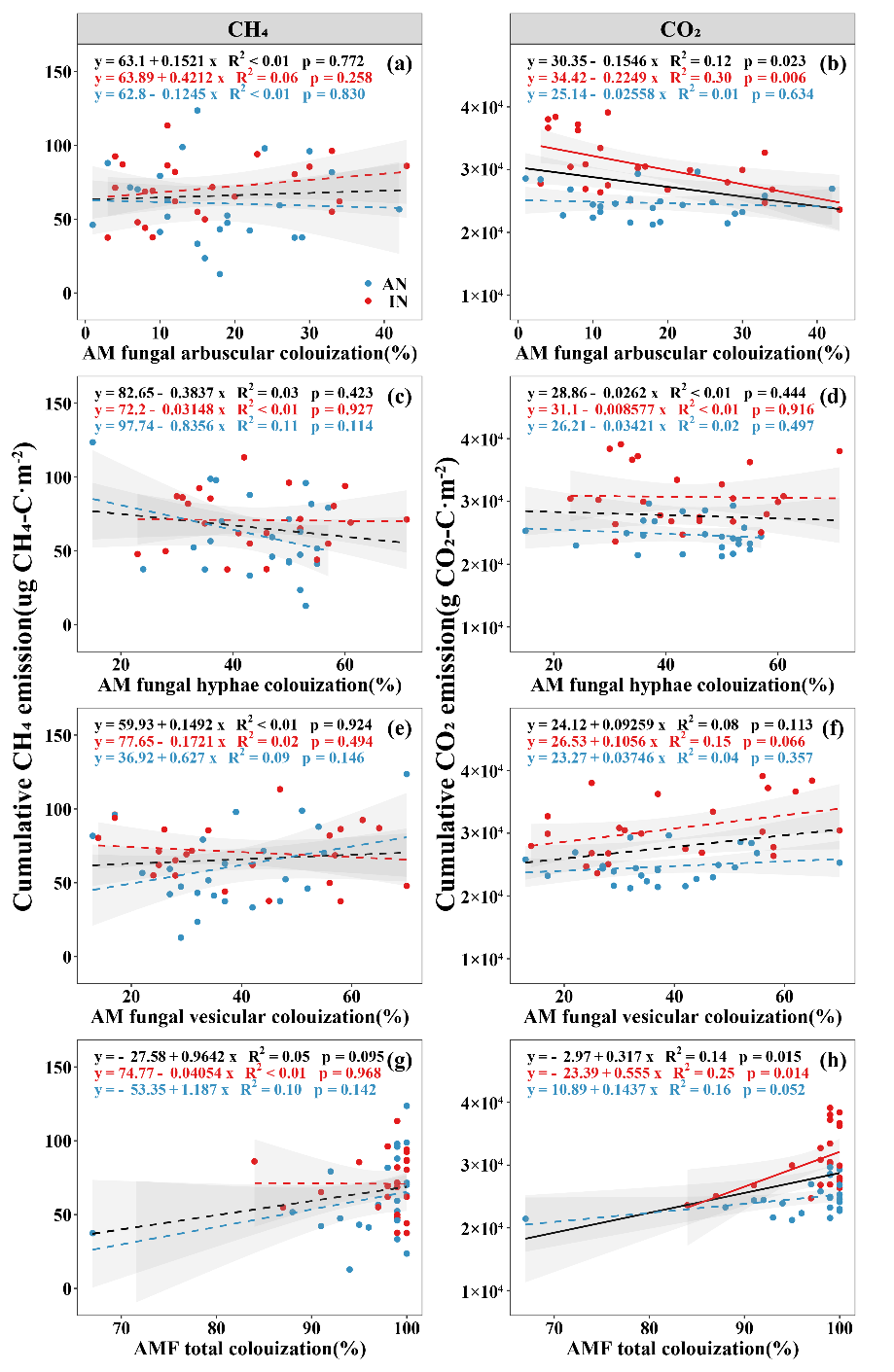


**FIGURE S4 Linear regression analyses of the relationships between cumulative CH_4_ and CO_2_ emissions with AM fungal colonization parameters.** AM fungal arbuscular colonization (a and b), AM fungal hyphae colonization (c and d), AM fungal vesicular colonization (e and f), and AM fungal total colonization (g and h). The gray shaded area represents 95% confidence intervals. Significant linkages are shown by solid lines and non-significant linkages are shown by dashed lines. Significant level: p<0.05.


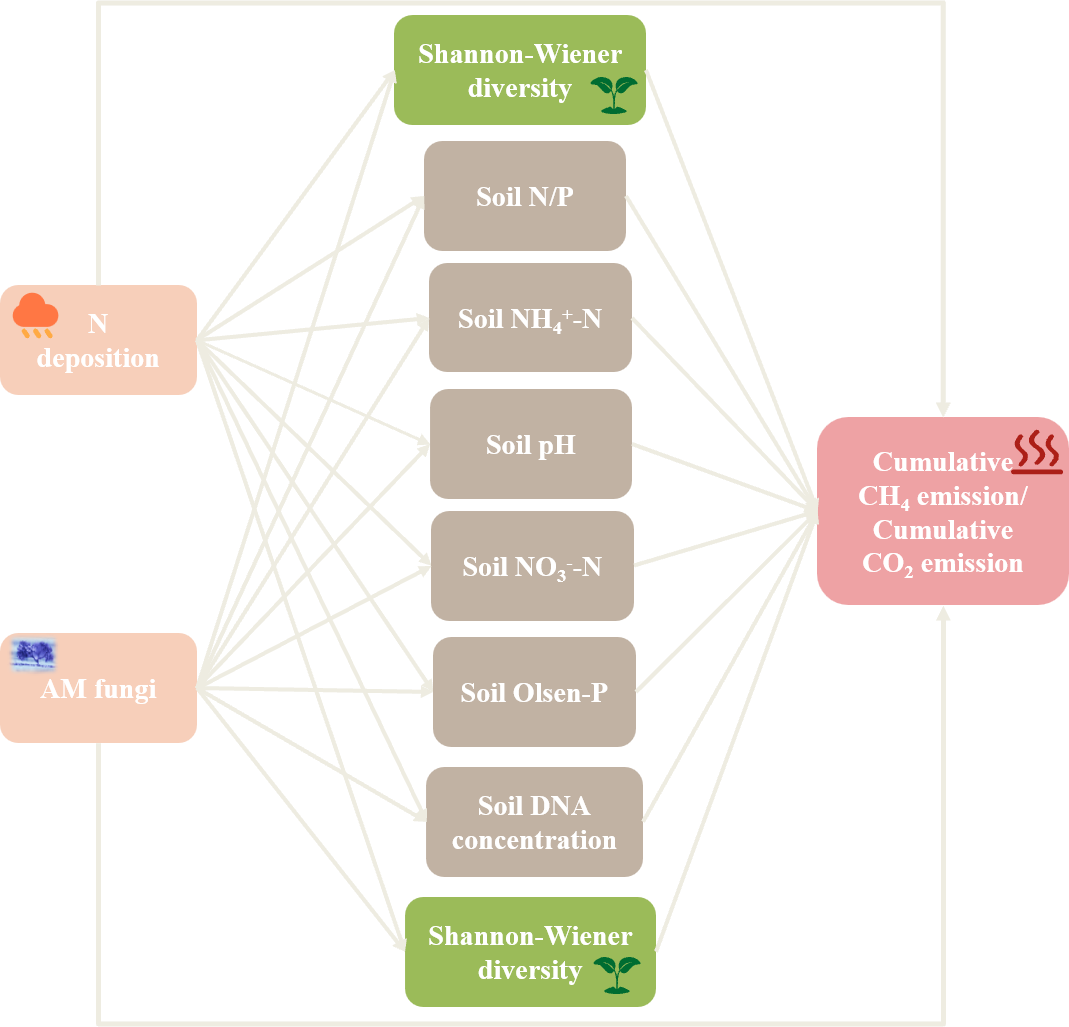


**FIGURE S5** The priori structural equation model (SEM) to reveal the potential influent pathways of AM fungi and increased N deposition on cumulative CH_4_ emissions through soil parameters (i.e., pH, NH_4_^+^-N, NO_3_^-^-N, Olsen-P, soil N/P), soil microbial biomass (soil DNA concentration), and plant community Shannon-Wiener diversity).
